# SPHK1 regulates proliferation and survival responses in triple-negative breast cancer

**DOI:** 10.18632/oncotarget.1874

**Published:** 2014-03-27

**Authors:** Arpita Datta, Ser Yue Loo, Baohua Huang, Lingkai Wong, Sheryl S.L. Tan, Tuan Zea Tan, Soo-Chin Lee, Jean Paul Thiery, Yaw Chyn Lim, Wei Peng Yong, Yulin Lam, Alan Prem Kumar, Celestial T. Yap

**Affiliations:** ^1^ Department of Physiology, Yong Loo Lin School of Medicine, National University of Singapore; ^2^ Department of Pharmacology, Yong Loo Lin School of Medicine, National University of Singapore; ^3^ Department of Biochemistry, Yong Loo Lin School of Medicine, National University of Singapore; ^4^ Department of Pathology, Yong Loo Lin School of Medicine, National University of Singapore; ^5^ Cancer Science Institute of Singapore, National University of Singapore; ^6^ Department of Chemistry, National University of Singapore, Singapore; ^7^ Department of Haematology-Oncology, National University Hospital, Singapore; ^8^ National University Cancer Institute, Singapore; ^9^ Institute of Molecular and Cell Biology (IMCB), A*STAR, Singapore; ^10^ School of Biomedical Sciences, Faculty of Health Sciences, Curtin University, Western Australia; ^11^ Department of Biological Sciences, University of North Texas, Denton, Texas, USA

**Keywords:** sphingosine kinase, breast cancer, chemotherapy

## Abstract

Triple-negative breast cancer (TNBC) is characterized by unique aggressive behavior and lack of targeted therapies. Among the various molecular subtypes of breast cancer, it was observed that TNBCs express elevated levels of sphingosine kinase 1 (SPHK1) compared to other breast tumor subtypes. High levels of SPHK1 gene expression correlated with poor overall and progression- free survival, as well as poor response to Doxorubicin-based treatment. Inhibition of SPHK1 was found to attenuate ERK1/2 and AKT signaling and reduce growth of TNBC cells *in vitro* and in a xenograft SCID mouse model. Moreover, SPHK1 inhibition by siRNA knockdown or treatment with SKI-5C sensitizes TNBCs to chemotherapeutic drugs. Our findings suggest that SPHK1 inhibition, which effectively counteracts oncogenic signaling through ERK1/2 and AKT pathways, is a potentially important anti-tumor strategy in TNBC. A combination of SPHK1 inhibitors with chemotherapeutic agents may be effective against this aggressive subtype of breast cancer.

## INTRODUCTION

Triple-negative breast cancer (TNBC) accounts for about 15% of breast cancer cases and is characterized by absence of estrogen receptor (ER), progesterone receptor (PR) and HER2 receptors. It is associated with aggressive histology and poor prognosis with shorter survival compared to other subtypes of breast cancers [1-3]. These tumors tend to occur more often in younger women and African American women [4]. Unlike other types of breast cancer, neither hormonal therapy such as tamoxifen or aromatase inhibitors, nor targeted therapy against HER2 using Herceptin (Trastuzumab), are effective against these neoplastic cells.

TNBC has a more aggressive clinical course than other subtypes of breast cancer [5]. They are currently treated with non-target specific chemotherapy such as Doxorubicin, 5-Fluorouracil (5-FU) and Docetaxel [6]. Doxorubicin is an anthracycline drug used in patients with metastatic breast cancer [7] but concerns include its relatively low therapeutic index and toxicities such as myelosuppression, immunosupression and cardiotoxicity [8]. 5-FU is also a widely used chemotherapeutic agent but is associated with cardiotoxicity, neutropenia and mucositis [9]. Docetaxel is a microtubule-stabilizing taxane that has higher antitumor activity compared to paclitaxel [10] but has been reported to cause toxicities such as neutropenia and fluid retention [11]. Taken together, treatment options for triple negative breast cancer remain limited and effective targeted therapies have yet to be developed [6].

Sphingosine kinases (SPHKs) are becoming established as critical mediators of oncogenesis [12]. Two isoforms of SPHK enzymes with distinct functions, SPHK1 and SPHK2, have been discovered [13, 14]. SPHK1, in particular, has been implicated in oncogenic roles in cancer, including proliferation, resistance to apoptosis and transformation [15]. Recent work in our laboratory revealed a positive correlation between SPHK1 and advanced tumor progression and SPHK1 as a predictor for mortality in colon cancer patients [16]. SPHKs convert sphingosine to S1P, which acts as an intracellular second messenger and extracellular ligand for specific receptors [17, 18]. Secreted S1P in turn, acts as a ligand for the family of G protein-coupled S1P receptors 1 to 5 (S1PR_1_ to S1PR_5_), and regulates a wide range of biological effects including transformation and migration [19].

Increased expressions of SPHKs have been observed in breast tumors [20] and microarray analysis suggests that breast cancer patients with high levels of the SPHK1 isoform are predisposed to poorer outcomes [21]. Most studies to date have highlighted the roles of SPHK1 in ER-positive breast cancer and its interactions with the estrogen receptor. SPHK1/S1P signaling has been reported to confer endocrine drug resistance and also promote estrogen-dependent tumorigenesis, motility and survival of ER-positive breast cancer [22, 23]. However, despite previous evidence that higher SPHK1 levels are found in ER-negative tumors [21], the therapeutic implications of SPHK1 in TNBC have not been well explored.

In this study, we examine the prognostic significance of SPHK1 in breast cancer and address the therapeutic potential of targeting SPHK1 in TNBCs, using SPHK1 knockdown experiments or treatment with a pharmacological SPHK1 inhibitor, 2,2-dimethyl-4S-(1-oxo-2-hexadecyn-1-yl)-1,1-dimethylethyl ester-3-oxazolidinecarboxylic acid (SKI-5C). Therapeutic effects of targeting SPHK1 on cell proliferation and cell death are evaluated upon SPHK1 inhibition alone or in combination with chemotherapeutic drugs.

## RESULTS

### Upregulated SPHK1 expression in human breast cancer tissues

**Figure 1:**
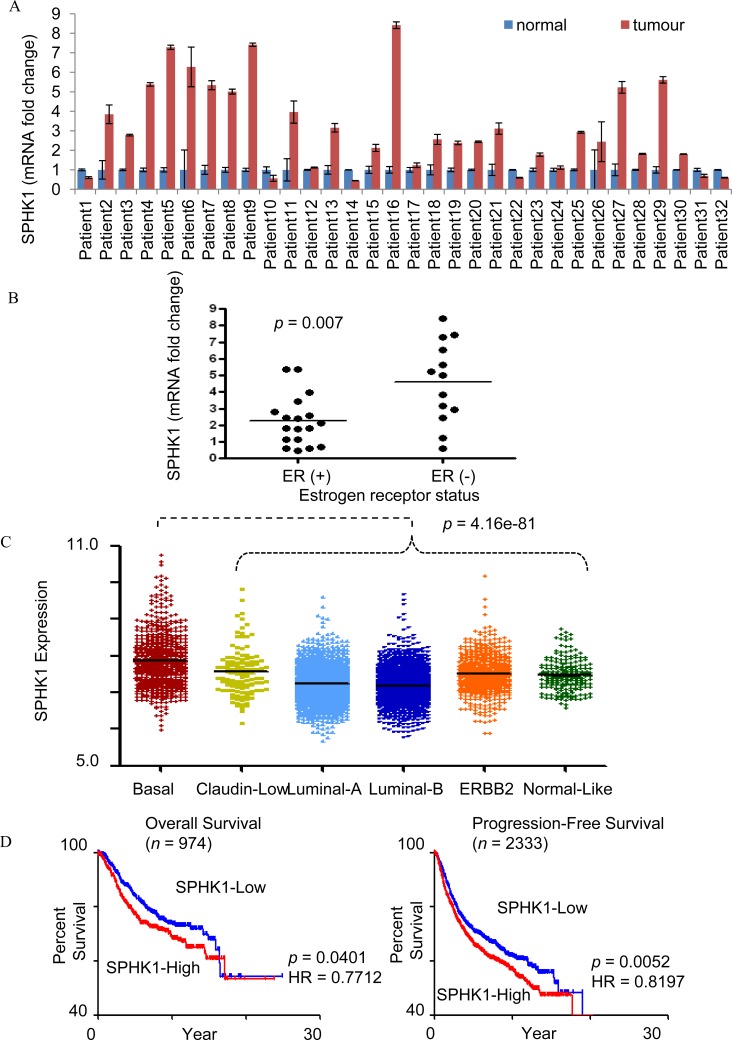
SPHK1 expression in breast tumors (A) SPHK1mRNA expression in human breast tumors and paired adjacent normal breast tissues by real-time PCR. Expression levels were normalized with GAPDH. A two-tailed Student t-test was used to calculate statistical significance. (B) SPHK1 mRNA expression in ER-positive, ER (+), and ER-negative, ER (-) cases. Each dot corresponds to an individual patient's fold change in relative SPHK1 mRNA levels between tumor and adjacent normal tissue. ER (-) patients showed significantly higher expression of SPHK1 than ER (+) patients (*p* = 0.007). (C) SPHK1 gene expression levels in breast cancer subtypes. Basal subtype has the highest SPHK1 gene expression (Mann Whitney Test, *p* = 4.16e−81), whereas Luminal-A and –B subtypes have the lowest SPHK1 gene expression (Mann Whitney Test, *p* = 1.1e−21, and *p* = 6.74e−37, respectively). (D) SPHK1 gene expression correlates with poor overall (left) and progression-free survival (right). Kaplan-Meier plots of overall and progression-free survival in all samples. Median expression was used to define SPHK1-Low and SPHK1-High. The *p*-value shown was computed by log-rank test. HR indicates the hazard ratio, and *n* in parentheses indicates number of samples.

We performed comparative analysis of SPHK1 expression using real time polymerase chain reaction (RT-PCR) on breast tumor tissues and adjacent normal breast tissues of 32 patients. The expression of SPHK1 mRNA was determined to be at least two folds higher in the breast tumor tissues compared to normal breast tissues in 62.5% of patients (20 of 32 cases) of (Fig. 1A). We observed that ER-negative tumors express higher SPHK1 mRNA levels than ER-positive tumors (p=0.007, Fig. 1B). Clinical data eg. grade, histology, ER/PR status of the patients can be found in Supplementary Data Table 1.We also found that triple-negative tumors express higher SPHK1 mRNA compared to other breast tumors (p = 6.98801E-06, Supplementary Data Fig. 1A). Through gene expression analyses, breast cancer tumors have been classified into six molecular tumor subtypes based on hierarchical clustering (basal-like, claudin-low, luminal A, luminal B, HER2-enriched, normal-like), where triple-negative tumors have been reported to be largely basal-like [24]. Using human breast cancer microarray data from Affymetrix U133A and U133Plus2 platforms, we found that basal-like subtype exhibits the highest SPHK1 gene expression among the various molecular subtypes (Fig. 1C). Mean and median values of SPHK1 gene expression are shown in Supplementary Data Table 2. Pair-wise comparison also shows significantly higher SPHK1 levels in the basal-like subtype compared to other molecular subtypes (Supplementary Data Table 3).

When patients from a breast cancer cohort were categorized into SPHK1-low and high groups based on median expression of SPHK1 (7.34 for OS, and 7.32 for PFS) (Supplementary Data Fig. 2A), patient survival analysis revealed an inverse correlation between SPHK1 expression level and the overall (OS) and progression-free survival (PFS) of breast cancer patients (p=0.0401 and p=0.0052 respectively) (Fig. 1D). Among the SPHK1-low and -high groups analysed for OS and PFS, it was found that the proportion of basal breast carcinoma patients in the SPHK1-high group was two to three times greater than the proportion of basal breast carcinoma patients in the SPHK1-low group (Supplementary Data Fig. 2B). Taken together, these observations suggest that high levels of SPHK1 expression are associated with poorer prognosis and lower survival in breast cancer.

### Upregulated SPHK1 expression in human breast cancer cell lines

**Figure 2:**
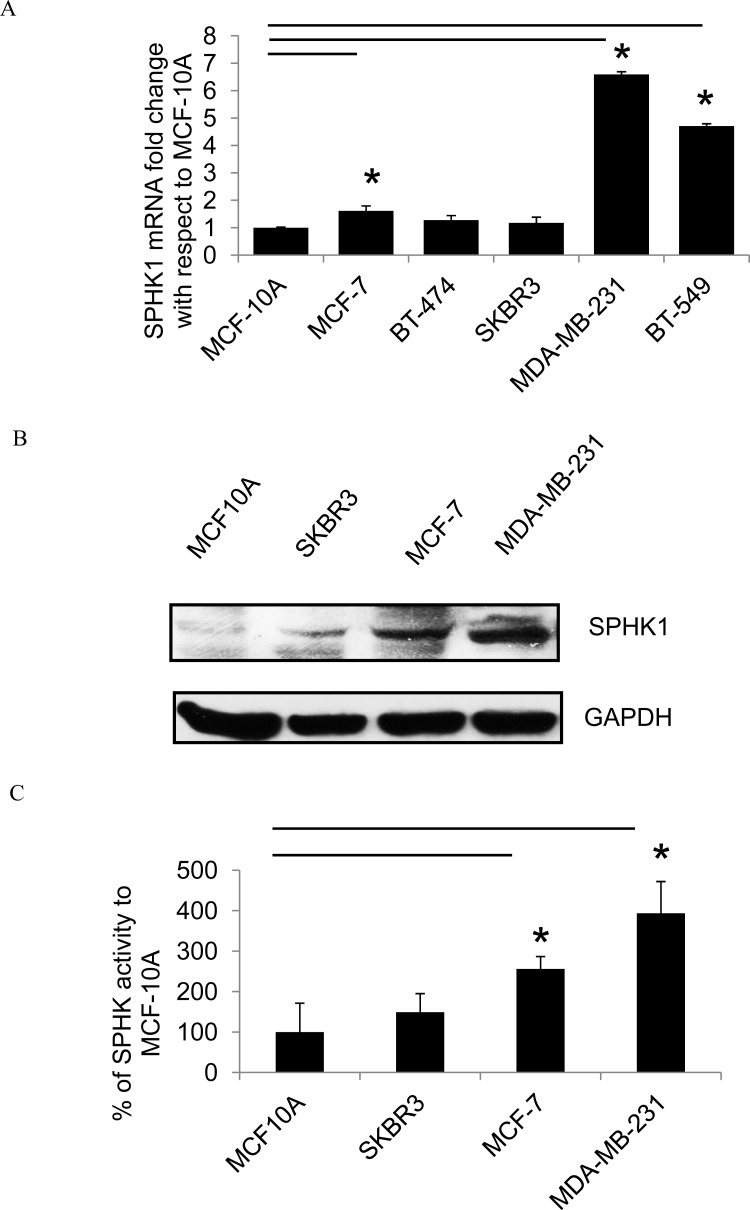
SPHK1 expression in breast cancer cells (A) SPHK1 mRNA levels by real-time PCR in breast cancer cell lines and the breast epithelial cell line MCF10A (normalized to GAPDH). The data is expressed as the fold change in SPHK1 expression compared to MCF-10A. Data represents the mean ± SD of at least three independent experiments; *p < 0.05. (B) The protein expression of SPHK1 in breast cancer cell lines was determined by Western blot. (C) Endogenous SPHK activity in cell lines. Data represents the mean ± SD of at least three independent experiments; *p < 0.05.

To determine SPHK1 expression in human breast cancer cell lines, real-time PCR and Western blots were performed on samples derived from five breast cancer cell lines and a breast epithelial cell line (MCF-10A). Real-time PCR showed that SPHK1 mRNA expression was higher in breast cancer cell lines compared to MCF-10A (Fig. 2A). Both SPHK1 protein expression and SPHK1 activity were also higher in breast cancer cell lines compared to MCF-10A (Fig. 2B-C). Among the breast tumor cell lines, the metastatic and triple-negative MDA-MB-231 cells exhibited the highest SPHK1 protein expression and the most pronounced SPHK1 activity. On the contrary, while there was no significant difference in SPHK2 mRNA expression in breast cancer patient data (Supplementary Data Fig. 1B), SPHK2 mRNA expression was lower in breast cancer cell lines compared to normal epithelial cell line MCF-10A (Supplementary Data Fig. 1C).

### Inhibition of SPHK1 activity induces apoptosis and inhibits proliferation of breast cancer cells

**Figure 3:**
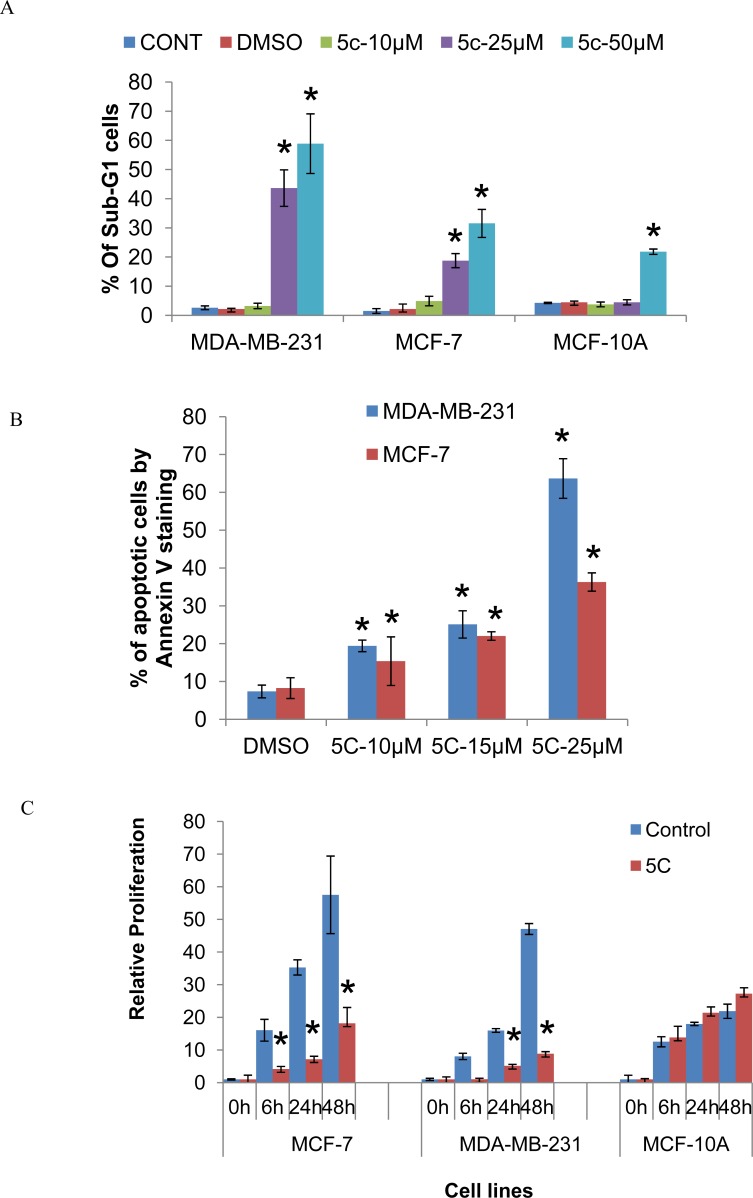
Inhibition of SPHK1 activity induces apoptosis and inhibits proliferation of breast cancer cells (A) Flow cytometric analysis of PI staining in MDA-MB-231, MCF-7 and MCF-10A cell lines fixed after treatment with 10-50μM SKI-5C for 24 hours. The graph shows percentages of cells in the Sub-G1 fraction for MDA-MB-231, MCF-7 and MCF-10A. Data represents the mean ± SD of at least three independent experiments; *p < 0.05. (B) MDA-MB-231 and MCF-7 cells were treated with 10-25μM SKI-5C for 24 hours and apoptotic cells were detected by flow-cytometric analysis of Annexin V staining. Data represents the mean ± SD of at least three independent experiments; *p < 0.05. (C) The effect of SKI-5C on cell proliferation was determined by culturing MCF-7, MDA-MB-231 and MCF10A cells under standard culture conditions in the presence of 10μM SKI-5C for 6 to 48 hours by BrdU assay. The relative fold changes in O.D. at 450nm compared to cells at the start of the assay (‘0’ hours) are shown, indicated as ‘Relative Proliferation’ on the y-axis. Data represents the mean ± SD of at least three independent experiments; *p < 0.05.

We investigated the effects of inhibiting SPHK1 activity using the SPHK1 pharmacological inhibitor, SKI-5C, on the cell cycle kinetics of triple-negative MDA-MB-231 and ER-positive MCF-7 cells. Cell cycle analysis by PI staining of MDA-MB-231 cells indicated that the induction of growth arrest by 10μM SKI-5C was associated with the accumulation of cells in sub-G1 phase. At higher doses of 25uM and 50uM SKI-5C, cell death was predominant with increases in the sub-G1 population to 43.05% and 50.98% respectively. MCF-7 cells also induced a dose-dependent increase in the sub-G1 population upon exposure to SKI-5C (Fig. 3A and Supplementary Data Fig. 3A). Significant increases in the percentages of sub-G1 populations were observed in both MDA-MB-231 and MCF-7 cells at 25μM and 50μM SKI-5C. In contrast, normal epithelial MCF-10A cells were less sensitive to SKI-5C, with no significant increases in sub-G1 population at doses lower than 50μM SKI-5C (Fig.3A). We further investigated the effects of SKI-5C on induction of apoptosis by Annexin V staining. There was a dose-dependent increase in apoptosis of MDA-MB-231 and MCF-7 cells within 24h, with 63.29% of MDA-MB-231 cells and 35% of MCF-7 cells undergoing apoptosis when treated with 25μM SKI-5C (Fig. 3B). These data indicate that the survival of breast cancer cells is significantly compromised by SPHK1 inhibition.

In addition, the effects of SPHK1 inhibition by SKI-5C on the proliferation of breast cancer cells were examined by BrdU assay. 10 μM of SKI-5C inhibited cell proliferation of both triple-negative MDA-MB-231 and ER-positive MCF-7 cells within 48 hours, but did not affect normal breast epithelial MCF-10A cells (Fig. 3C). We also compared the use of SKI-5C (SPHK1-specific inhibitor) and DMS (N,N-dimethyl-sphingosine) (a SPHK1 and SPHK2 dual-inhibitor). We observed that SKI-5C was more effective in inhibiting proliferation of MDA-MB-231 as its baseline SPHK1 level is high. In MCF-7 and MCF-10A cell lines, which have lower levels of SPHK1 expression, the inhibition of cell viability by SKI-5C and DMS were not significantly different. In addition, MCF-10A cells that have high SPHK2 levels, responded to SKI-5C and DMS treatment equally, suggesting that inhibition of SPHK2 had little or no effect on cell viability (Supplementary Data Fig. 4A) These results indicate that the inhibition of SPHK1 activity by SKI-5C effectively impairs the growth potential of breast cancer cells.

### Inhibition of SPHK1 activity impairs colony formation and breast tumor formation of MDA-MB-231 cells *in vivo*

**Figure 4:**
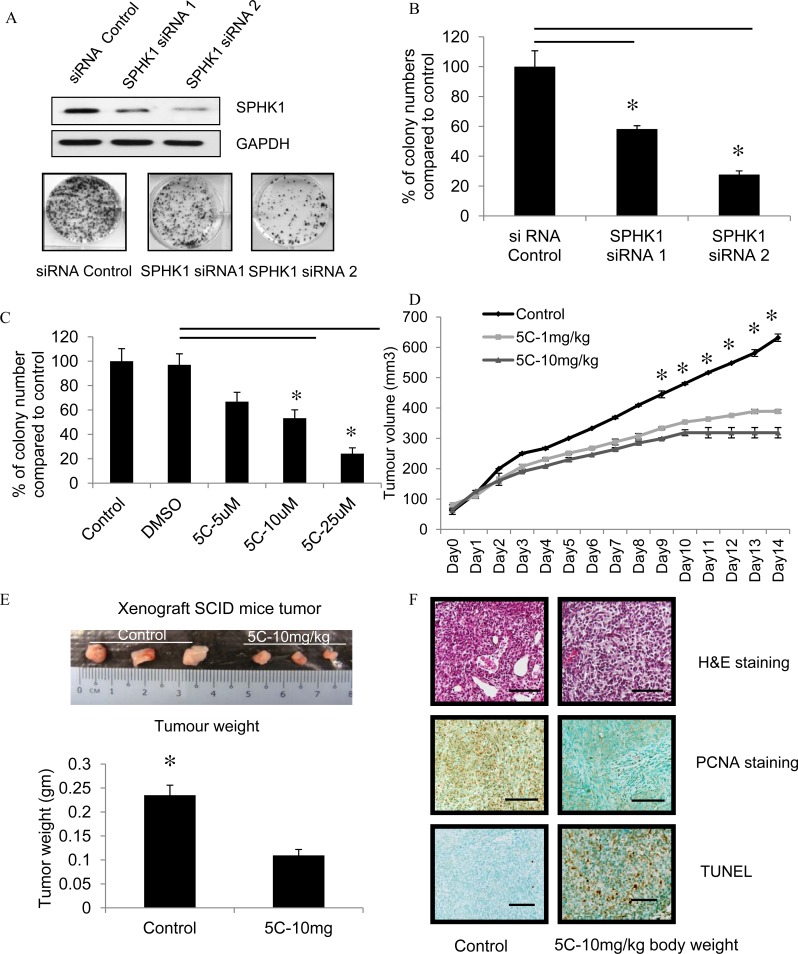
Inhibition of SPHK1 activity impairs colony formation and breast tumor formation by MDA-MB-231 cells in immunodeficient mice (A) Top: Representative western blot of SPHK1 and GAPDH in MDA-MB-231 after transfection with SPHK1-specific siRNAs (SPHK1 siRNA1 and siRNA2) or control siRNA for 48 hours. Bottom: Representative images of colony formation assay in MDA-MB-231 cells transfected with either control siRNA or SPHK1 siRNAs. 48 hours after transfection, equivalent numbers of live cells were replated, cultured for 7 days, then stained with crystal violet. (B) Number of colonies from (A) were counted. Data are expressed as mean ± S.D. of triplicate measurements and of three independent experiments; *p < 0.01. (C) The effect of SKI-5C on colony formation was determined by treating MDA-MB-231 breast cancer cells grown at low cell density (1000 cells/well in a 6-well plate) with 5 - 25μM SKI-5C for 7 days. Colonies were stained with crystal violet and quantified. Data are expressed as mean ± S.D. of triplicate measurements and of three independent experiments; *p < 0.01. (D) SCID mice (6 mice per group) with palpable MDA-MB-231 tumors (2 tumors per mouse) were injected intraperitoneally with saline (control) or SKI-5C 1 or 10mg/kg body weight for 14 consecutive days. Tumor volumes were recorded daily. Animals were euthanised and tumors excised. (E) Representative picture shows MDA-MB-231 tumors treated with saline control or with SKI-5C at 10mg/kg body weight. Animals were euthanised and tumors excised and weighed. SKI-5C inhibits tumor growth and reduces tumor weight in a dose-dependent manner. Data are expressed as mean ± S.D. (F) Tumor histology. Paraffin-embedded tumor sections were stained with either H&E or PCNA. Apoptotic cells were visualized by TUNEL staining and counterstained with methyl green. Slides were analyzed by fluorescence microscopy. Representative section from n=3 individual treated tumors. Scale bar represents 50 μm.

We investigated the effects of down-regulating SPHK1 levels by siRNA knockdown in the triple-negative MDA-MB-231 on colony formation ability. For this purpose, two siRNA constructs that target SPHK1 were used in triple-negative MDA-MB-231 cells (SPHK1 siRNA 1 and SPHK1 siRNA 2), and their effects compared to control siRNA. SPHK1 protein was reduced more effectively by SPHK1 siRNA 2 compared to siRNA1, which corresponded to significant reductions in the number of colonies formed compared to cells transfected with control siRNA (Fig. 4A-B).

We next determined the effects of the SPHK1 pharmacological inhibitor SKI-5C on the colony formation ability of breast cancer cells. Triple-negative MDA-MB-231 cells were treated with SKI-5C in concentrations of 5-25μM for 7 days and colony formation was measured. There was a significant dose-dependent reduction in the number of colonies formed by SKI-5C-treated cells compared to control cells (Fig. 4C). These results indicate that the inhibition of SPHK1 activity by siRNA silencing or pharmacological inhibitor SKI-5C effectively impairs the growth potential of TNBC cells. We also investigated the effects of down-regulating SPHK2 levels by SPHK2 siRNA. Results showed that SPHK2 siRNA knockdown in breast cancer cell did not affect colony formation and cell proliferation (Supplementary Data Fig. 5A-C). This provides further evidence that SPHK2 is not likely to have significant effects on growth and survival of breast cancer cells.

To determine the therapeutic potential of pharmacologic inhibition of SPHK1 in TNBC, the effects of SKI-5C on tumor formation by MDA-MB-231 cells were examined using a xenograft model in immunodeficient mice. MDA-MB-231 cells were injected subcutaneously and bilaterally into the flanks of SCID mice [25]. Treatment with daily intra-peritoneal injections of SKI-5C at 1 and10mg/kg was instituted from day 7 for 14 consecutive days, and compared with control mice injected with saline. Treatment with SKI-5C significantly decreased tumor growth compared to control (Fig. 4D). After 14 days, the mean volume of the MDA-MB-231 tumors, in mice treated with 10mg/kg SKI-5C, was less than 50% of the tumors in the control mice (control group mean = 631mm^3^ ±12.297, SKI-5C-10mg/kg body weight = 318mm^3^ ±17.125; P = 1.01568E−11). After 14 days of treatment, all mice were sacrificed and the subcutaneous tumors were extracted and weighed. Tumor weights of SKI-5C-treated mice were significantly lower than the control mice (Fig. 4E). We also examined the tumors by histological analysis. Tumors from control mice stained strongly for proliferating cell nuclear antigen (PCNA), indicating high proportions of proliferating cells in control tumors compared to SKI-5C-treated tumors. In contrast, immunohistochemical analysis of tumors from SKI-5C treated mice revealed a high proportion of apoptotic cells compared to control tumors as determined by nuclear fragmentation (TUNEL staining) (Fig. 4F). In summary, our results indicate that inhibition of SPHK1 is effective in suppressing the growth of triple-negative breast tumors *in vitro* and *in vivo*, suggesting that SPHK1 could be a useful therapeutic target in TNBC.

### Serum induces pro-survival signals via SPHK1

**Figure 5:**
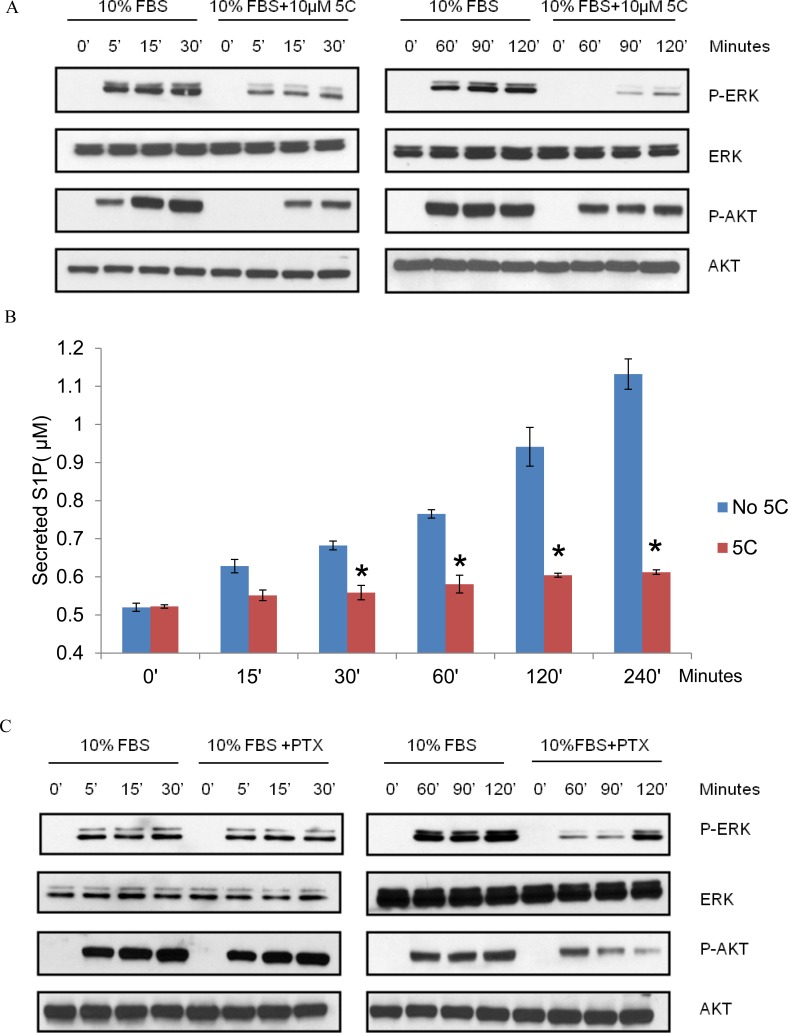
SKI-5C inhibits the serum-dependent activation of ERK1/2 and AKT signalling and S1P export Western blots showing the effect of SKI-5C on serum-dependent activation of ERK1/2 and AKT in MDA-MB-231 cells stimulated with 10% FBS for 5 - 120 minutes after treatment with 10μM SKI-5C. Control cells were stimulated with 10% FBS after pretreatment with DMSO. (B) Secreted S1P levels determined by ELISA in MDA-MB-231 cells pretreated with 10μM SKI-5C, followed by stimulation with 10% FBS for up to 240 minutes. Control cells were pretreated with DMSO. Data are expressed as mean ± S.D. of triplicate measurements and of three independent experiments; *p < 0.01. (C) Representative Western blot (of three independent experiments) showing the effect of PTX on serum induced ERK1/2 and AKT phosphorylation.

The extracellular signal-regulated kinases (ERK1/2) and PI3-kinase/AKT are major pathways regulating key cellular processes including proliferation, cell cycle progression and cell survival. 10% FBS was used to stimulate SPHK1 activity and examine the SPHK1-dependent mechanisms induced by serum for breast cancer cell survival. Treatment of triple-negative MDA-MB-231 cells with SKI-5C caused a rapid and sustained decrease in serum-induced phosphorylation of both ERK1/2 and AKT and SKI-5C inhibited early as well as sustained activation of ERK1/2 and AKT (Fig. 5A). The regulation of both ERK1/2 and AKT activation in response to serum stimulation could represent potential mechanisms by which SPHK1 promotes survival in breast cancer cells.

The biological functions of SPHK1 relies primarily on its product, S1P, which functions as an intracellular second messenger and ligand for the G protein-coupled S1P receptors [26]. We found that secreted S1P levels were significantly increased within 30 minutes of serum stimulation in MDA-MB-231 cells, and this increase was inhibited by 10μM SKI-5C (Fig. 5B). This indicates that SPHK1 activation is required for S1P production and release from the serum-stimulated cells.

Activation of ERK1/2 and AKT by agents that act through G protein-coupled receptors is often mediated by G_i_ proteins, and is therefore sensitive to pertussis toxin (PTX), which inhibits the function of G_i_ proteins [26]. To ascertain if S1P receptors are involved in serum-dependent ERK1/2 and AKT phosphorylation, MDA-MB-231 cells were stimulated with 10% FBS in the presence of PTX (100ng/ml for 2 hours). PTX inhibited the sustained activation of ERK1/2 and AKT by serum stimulation but did not affect the early activation of both pathways (Fig. 5C). These findings suggest that the sustained activation of ERK1/2 and AKT by serum requires S1P secretion and S1P-receptor stimulation.

### SPHK1 inhibition enhances efficacy of chemotherapeutic treatment in breast cancer cells

**Figure 6:**
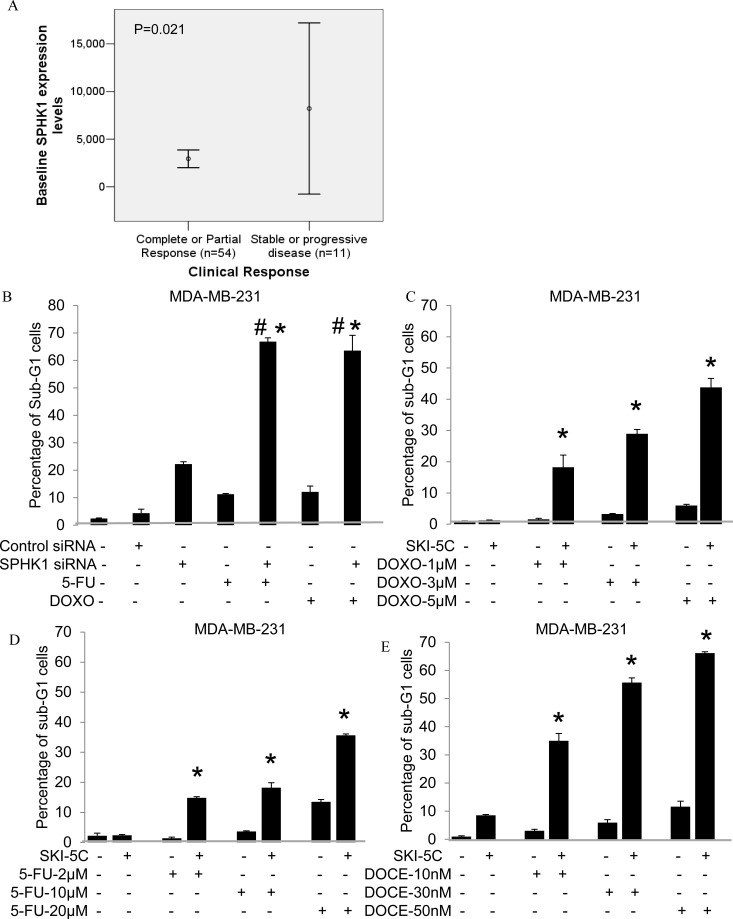
SPHK1 affects breast cancer sensitivity to Doxorubicin and 5-FU (A) Comparison of baseline SPHK1 levels between clinical non-responders and responders to Doxorubicin-based treatment (8212±14901 vs 2948±3411, p=0.021). (B) Percentages of sub-G1 population by PI analysis in cells transfected with either control siRNA or SPHK1 siRNA1 for 48 hours (*p* = 0.00024), followed by addition of 20μM 5-FU or 10μM Doxorubicin for 24 hours. Data are expressed as mean ± S.D. of triplicate measurements and of three independent experiments; *p < 0.05 compared to SPHK1 siRNA 1, # *p* < 0.05 compared to 5-FU or Doxorubicin (Student's t- test). (C-E) Sub-G1 populations by PI analysis in combination treatment of cells using 10μM SKI-5C with low doses (below IC_50_) of 1-10μM Doxorubicin or 2 - 20μM 5-FU or 10-50nM Docetaxel for 24 hours compared to treatment with either SKI-5C, Doxorubicin, 5-FU or Docetaxel alone. Data represents the mean ± SD of at least three independent experiments; *p < 0.05.

Microarray analysis was performed on 65 breast tumors from patients with locally advanced or metastatic cancers who were recruited into a prospective study on Doxorubicin and Docetaxel-based chemotherapy. We observed significantly higher SPHK1 mRNA levels in patients who were non-responders to treatment compared to complete or partial responders (p=0.021) (Fig. 6A). Non-responders achieved only stable or progressive disease as their best response (WHO criteria) after 6 cycles of chemotherapy. This suggests that SPHK1 may be a useful predictor of chemotherapy response, and may contribute to chemotherapy resistance.

We investigated the effectiveness of combining Doxorubicin and 5-FU with SPHK1 knockdown or inhibition by SKI-5C in ER-negative breast cancer cells. MDA-MB-231 cells transfected with SPHK1 siRNA or control siRNA were exposed to either 5-FU or Doxorubicin before being harvested for PI analysis by flow cytometry. Whilst silencing of SPHK1 in MDA-MB-231 cells induced a small but significant increase in cell death of about 20%, the combination with 5-FU or Doxorubicin induced further synergistic increases in cell death of about 60% compared to treatment with either drug alone (20% cell death) (Fig. 6B).

The combination of SKI-5C with Doxorubicin, 5-FU and Docetaxel used at doses below IC_50_ (Supplementary data Fig. 6A) was also examined. When MDA-MB-231 cells were pre-treated with 10μM SKI-5C, cell death was significantly enhanced, with synergistic increases of up to 43.8% cell death with 10μM Doxorubicin, 35% cell death with 20μM 5-FU and 66% cell death with 50nM Docetaxel (Fig. 6C-E). The combination of SKI-5C and Doxorubicin or Docetaxel was also carried out in another triple negative breast cancer cell line Hs578T, which similarly yielded significant increases in cell death compared to each drug alone (Supplementary Data Fig. 6B-C).

## DISCUSSION

Numerous studies have shown that SPHK1 is critical for growth, metastasis and chemo-resistance of human breast cancers [27]. Previous work in breast cancer has shown that SPHK1 is activated by estrogen signaling and promotes estrogen-dependent oncogenesis [28, 29]. However, the basal molecular subtype of breast cancers, which often lack the ER receptor and are thus unresponsive to anti-estrogens, have been found to express high baseline expression levels of SPHK1. This subtype of breast cancers has been reported to be more aggressive and bear a poorer survival outcome. Therefore, this study is focused on evaluating SPHK1 as a potential therapeutic target for treatment of basal breast carcinoma patients. On the other hand, the role of SPHK2 in cancer is still largely unknown. It has been reported that over-expression of SPHK2 decreases cell growth and enhances apoptosis [30], which seems to suggest that it may function as a tumor suppressor.

In our studies, up-regulation of SPHK1 in TNBC was identified by several lines of evidence, including comparative determination of SPHK1 expression in breast cancer tissues and cell lines, as well as microarray data of human breast cancers from Affymetrix U133A and U133Plus2 platforms. Our study has provided evidence that SPHK1 up-regulation plays important roles in the growth and survival of TNBC cells. Furthermore, our microarray analysis revealed significantly elevated levels of SPHK1 in poor responders to Doxorubicin/Doxetaxel-based chemotherapy, showing that SPHK1 may play a critical role in breast cancer resistance to chemotherapy.

In TNBC cells, SKI-5C reduced serum-induced growth and survival mediated through ERK1/2 and AKT pathways, indicating that TNBC cells are sensitive to SPHK1 inhibition. We show that activation of these pathways in response to serum is executed by different SPHK1-dependent mechanisms. The activation of ERK1/2 and AKT signaling depends on both the intracellular S1P production by SPHK1, as well as the SPHK1-dependent secretion of S1P acting on G protein-coupled receptors which functions to sustain ERK1/2 and AKT signaling. Recently, SPHK1 was shown to increase S1P export from ER-positive breast cancer cells in response to estrogen [31]. In ER-positive breast cancer patients, recent evidence also indicates that the activation of SPHK1 and SPHK1-mediated pathways, including S1P and ERK1/2, is associated with endocrine resistance and earlier recurrence [32]. Our data indicates that SPHK1 activity is also crucial for S1P export from TNBC cells in response to serum stimulation. As both early and sustained phases of ERK1/2 and AKT signaling are sensitive to SKI-5C, SPHK1 inhibitors are effective against proliferative responses to intracellular S1P, as well as secondary stimulation from S1P secretion. There is evidence that activated ERK1/2 can also phosphorylate and activate SPHK1 [33]. It is thus possible that a positive feedback loop exists, whereby activated ERK1/2 stimulates SPHK1, leading to increased intracellular S1P as well as S1P export. Exported S1P then in turn stimulates ERK1/2 via S1P receptors, leading to sustained activation of both ERK1/2 as well as SPHK1. As both ERK1/2 and SPHK1 have been implicated in several oncogenic pathways, the inhibition of SPHK1 by SKI-5C may effectively down-regulate multiple molecular pathways in breast cancer by its potential to interrupt this positive feedback loop. Therefore, these data provide further evidence for the pharmacological inhibition of SPHK1 as a potential therapeutic strategy in the treatment of TNBC.

*In vitro*, SPHK1 siRNA knockdown enhanced the cytotoxic effects of Doxorubicin and 5-FU in TNBC cells. This is consistent with our observations of elevated SPHK1 in patients who respond poorly to Doxorubicin. Our data suggests that the combination of SPHK1 inhibitors with conventional chemotherapeutic drugs could be effective against TNBCs. Indeed, levels of tumor cytotoxicity observed using low doses of Doxorubicin, 5-FU and Docetaxel were appreciably enhanced by combination with SKI-5C. This is supported by emerging evidence in other cancers showing the enhanced cytotoxicity of conventional chemotherapeutic agents upon inhibiting SPHK1 [16, 34, 35]. To extend the clinical application of this strategy, breast cancer patients who exhibit poor response to Doxorubicin-based chemotherapy may benefit from SPHK1-targeted therapy as the significantly higher levels of SPHK1 in their tumors may be conferring tumor-survival advantages.

In summary, our studies indicate that SPHK1 is a potential therapeutic target not only in ER-positive breast cancers, which have been previously reported, but also TNBCs. We validated the higher expression of SPHK1 mRNA levels in triple-negative human breast tumors compared to receptor-positive tumors, which correlate with poor overall and progression-free survival in breast cancer patients. Importantly, we also observed that poor patient response to Doxorubicin-based treatment is significantly associated with high SPHK1 levels. Using xenograft and *in vitro* models, we demonstrate that inhibition of SPHK1 in TNBC cells reduces growth and attenuates signaling mechanisms in TNBC cells through S1P, ERK1/2 and AKT pathways. Furthermore, SPHK1 inhibition sensitizes TNBC cells to chemotherapeutic agents such as Doxorubicin, 5-FU and Docetaxel. The development of SPHK1-targeted drugs should thus be further explored to improve the treatment and prognosis of patients with TNBCs.

## MATERIALS AND METHODS

### Materials

We synthesized 2,2-dimethyl-4S-(1-oxo-2-hexadecyn-1-yl)-1,1-dimethylethylester-3-oxazolidine carboxylic acid (SKI-5C), a specific inhibitor of SPHK1, as previously described [36]. SKI-5C is also available from Cayman Chemical (CAY10621). Doxorubicin, 5-FU and pertussis toxin (PTX) were purchased from Sigma. SPHK1 antibody was purchased from Exalpha Biologicals Inc; GAPDH antibody was from Santa Cruz Biotechnologies (CA, USA) and phospho-ERK1/2, ERK1/2, phospho-AKT, AKT antibodies were from Cell Signaling Technology Inc, USA.

### Cell lines and tissues

Human mammary and breast cancer cell lines including MCF10A, MCF7, MDA-MB-231, SKBr3, BT-474, BT-549 and Hs578T were obtained from American Type Culture Collection (ATCC, USA), and maintained at 37ºC with 5% CO_2_ in growth medium MEGM (Mammary Epithelial cell growth medium), DMEM (Dulbecco's Modified Eagle's Medium) and RPMI-1640 respectively supplemented with 10% fetal bovine serum (FBS). 32 human breast tumor tissues and adjacent normal breast tissues were obtained from NUH-NUS Tissue Repository for real-time PCR measurement of SPHK1 levels.

For serum stimulation, cells were plated in serum-free media in 6-well plates at a density of 10^5^ cells/well for 14 hours. Subsequently, cells were pretreated with inhibitors for 15 minutes before stimulation with 10% FBS. Samples were lysed in RIPA buffer (50mM Tris-HCL, pH7.4; 1% NP-40; 0.25% Na-deoxycholate; 150mM NaCl; 1mM EDTA) with protease and phosphatase inhibitors (Roche, USA). Lysates were used for Western blot analysis and measurement of sphingosine kinase activity.

### Analysis of SPHK1 expression in clinical therapeutics cohort

For the clinical therapeutics analysis, female patients with histologically or cytologically proven locally advanced or metastatic breast cancer were recruited into a prospective phase II study and randomized to one of two alternating sequences of Doxorubicin (A) and Docetaxel (T), starting either with Doxorubicin 75mg/m2 or Docetaxel 75mg/m2 every 3 weeks for six cycles (A-T-A-T-A-T, n=49; T-A-T-A-T-A, n=51). The study protocol was approved by the institutional ethics committee (National Healthcare Group Domain Specific Review Board), and all patients provided written informed consent. Clinical response was categorized according to the WHO criteria into complete response, partial response, stable disease and progressive disease after 6 cycles of chemotherapy. Tumor core biopsies were taken from each patient at baseline for immunohistochemical analysis for estrogen receptor and transcription analysis for SPHK1. Total tumor RNA was labelled by biotin and hybridized on the Affymetrix U133+2 microarray chip and analyzed according to the manufacturer's instructions (Santa Clara, California, USA), and tumor SPHK1 expression data (probe set 219257s_at) extracted for this analysis. The gene expression data for this study is publicly available at the Centre for Information Biology Gene Expression Database (CIBEX, http://cibex.nig.ac.jp/index.jsp; CIBEX Accession number: CBX91). The clinical trial was registered with clinicaltrials.gov (ClinicalTrials.gov ID: NCT00212082).

### Quantitative real-time PCR (RT-PCR)

Total RNA was extracted using the RNeasy Mini Kit (Qiagen), according to the manufacturer's protocol and 500ng of total RNA was converted to cDNA per 20μl reaction using ImProm-II^™^ Reverse Transcription System (A3803, Promega). Real-time PCRs were performed with an ABI Prism 7300 Real time PCR system (Applied Biosystems) using custom Taqman Gene Expression Assay. Data were normalized to GAPDH. A two-tailed Student t-test was used to calculate statistical significance.

### Data preprocessing of Affymetrix microarray for SPHK1 gene expression

Microarray data of human breast cancer on Affymetrix U133A or U133Plus2 platforms were downloaded from Array Express and Gene Expression Omnibus (GEO). In order for our study to be consistent with broader generalization and attain a larger sample size, we did not impose limit on age, stage, tumor grade or lymph node status. We included all primary or metastatic breast cancer patients (age > 20) found on GEO at the time the study is initiated. We checked the distribution of grade, ER status, HER2 status, lymph node status, and age in SPHK1-low and high groups in OS and PFS. The panel of human breast cancer data utilized for analysis comprises 3,992 tumor samples from 26 cohorts, including E-TABM-158 (n=130), GSE11121 (n=200), GSE12276 (n=204), GSE1456 (n=159), GSE1561 (n=49), GSE19615 (n=115), GSE20181 (n=176), GSE2034 (n=286), GSE21653 (n=266), GSE23177 (n=116), GSE23593 (n=50), GSE23988 (n=61), GSE25066 (n=508), GSE26639 (n=226), GSE31519 (n=67), GSE3494 (n=251), GSE3744 (n=47), GSE4922 (n=40), GSE5327 (n=58), GSE5460 (n=127), GSE5764 (n=10), GSE6532 (n=414), GSE6596 (n=24), GSE7390 (n=198), GSE9195 (n=77), and HESS cohort (n=133) [37]. Out of the 3,992 tumor samples, 974 have overall survival information, and 2,333 have progression-free survival information. Robust Multichip Average (RMA) normalization was performed on each dataset. The normalized data was combined and subsequently standardized using ComBat [38] to remove batch effect.

### Western blot analysis

Equal amounts of protein were separated by SDS-PAGE and transferred to polyvinylidene difluoride membranes (PVDF) (Millipore, USA). Blots were incubated with primary antibodies against SPHK1, GAPDH, phospho-ERK1/2, ERK1/2, phospho-AKT and AKT, followed by appropriate HRP-conjugated secondary antibodies. Immunocomplexes were visualized by ECL Western blot analysis detection system (Amersham Biosciences).

### Measurement of sphingosine kinase activity (fluorometric method)

The protocol has previously been established [39]. 70μg of protein was incubated with 20μM of 15-NBD-Sph (prepared as a complex with BSA) and ATP (1mM) in SPHK buffer (50mM 4-(2-hydroxyethyl)piperazine-1-ethanesulfonic acid, pH 7.4, containing 15mM MgCl_2_, 0.005% Triton X-100, 10mM KCl). After incubation for 30 minutes at 37°C, 100μl 1M potassium phosphate buffer (pH 8.5) was added, followed by 500μl CHCL3/MeOH 2:1. After brief mixing, phases were separated by centrifugation, the upper aqueous layer was removed and placed into polystyrene microplates (Greiner Bio-One), followed by addition of 75μl dimethylformamide (Merck). Fluorescence intensity was measured at 485/535nm.

### Cell proliferation, cell viability and colony formation

Cell proliferation was ascertained by plating cells at a density of 2000 cells/well in 96-well plates, then proliferation was measured using the BrdU proliferation assay (Roche). The optical densities (O.D) at 450nm for both untreated and treated cells measured at the start of the assay (‘0’ hours) were taken as 1. The relative fold changes in O.D. (450nm) for untreated and treated cells at 6, 24, and 48 hours were calculated against their respective ‘0 hour’ control cells, and expressed as ‘Relative Proliferation’. For cell viability assays, 10^4^ cells were plated in 96-well plates. Cell viability was measured using the WST-1 assay (Roche). The ability for colony formation at low cell density was determined by plating 1000 cells into 6-well plate and culturing for 7 days. Cells were subsequently stained with 0.5% crystal violet in 30% ethanol, 3% formaldehyde for 10 minutes at room temperature. Stained colonies were imaged and counted by Image J software.

### Cell death analysis

Cells (10^5^/well in 2ml) were grown overnight with 10% FBS, incubated with 5-50μM SKI-5C for 24 hours, fixed with 70% ethanol and stained with propidium iodide (0.1% Triton X-100, 200ug/ml RNase A, 20ug/ml propidium iodide in PBS). Detection of sub-G1 (dead) cells was performed using flow cytometry. For detection of apoptotic cells, surface expression of phosphatidylserine was assessed using Annexin-V–Fluos kit (Roche). SKI-5C-treated cells were re-suspended in PBS with FITC-conjugated Annexin-V. Flow cytometry analysis was conducted using the BD FACSCalibur and Cell Quest Pro Software.

### Measurement of S1P

Extracellular S1P levels were measured using S1P competitive ELISA with a sensitivity of 30nM S1P (Echelon Bioscience Inc). Cells (10^5^/well in 2ml) were plated without FBS for 14 hours, then pretreated with inhibitors for 15 minutes before stimulation with 10% FBS for up to 240 minutes. The supernatant was collected for S1P analysis by manufacturer's instructions.

### siRNA transfection

The siRNAs (Qiagen) were transfected into cells using Lipofectamine RNAi Max (Invitrogen), according to manufacturer's protocol. After 6 hours, transfection medium was removed and replaced with fresh complete medium. Cells were harvested after 48 hours of transfection.

### Tumor xenograft model

All experiments involving mice were conducted in accordance with NUS Institutional Animal Care and Use committee (Approved IACUC Protocol No: 108/06A409) guidelines which approves the use of animal (mice) for research. Female severe combined immunodeficient (SCID) mice (weighing 7 to 8 gm) aged 8 to 10 weeks, were obtained from Biological Resource Centre (BRC) Agency for Science, Technology and Research (A^*^ STAR) Biopolis. MDA-MB-231 cells (2 × 10^6^ suspended in 100 μL sterile PBS) were injected subcutaneously into 2 sites on both flanks of SCID mice and allowed to grow for 6 days. To determine the size of the subcutaneous tumors, we used the ellipsoid volume formula [40, 41], an accurate estimation of actual tumor volume [42] which has been applied by other laboratories in several breast cancer xenograft models [43, 44]. Tumor measurements were made daily with calipers, and tumor volume was calculated using the formula: (π × [length in millimetres] × [width in millimeters^2^]/6. On day 7, mice were randomly assigned to different groups that were injected intra-peritoneally (i.p.) with 100 μl saline (control) (n=6) and SKI-5C at dose of 10mg/kg BW (n=6) for 14 consecutive days. At the end of the experiment, the animals were sacrificed and tumors were removed for measurement of tumor weight and fixed in formalin, and embedded in paraffin. Sections were de-paraffinised and rehydrated and were stained with hematoxylin-eosin or with antibodies against proliferating cell nuclear antigen (PCNA) (DakoCytomation). Apoptotic cells were detected by ApopTag Peroxidase in situ Apoptosis Detection Kit (Chemicon international). Staining was visualized using DAB (DakoCytomation) and counter-stained by methyl green.

### Statistical Analysis

The statistical significance of difference was evaluated using the unpaired Student's t-test. The tests were two-sided and level of significance was set at *p <0.05.

## SUPPLEMENTARY FIGURES AND TABLES




