# Supplementary material for: SPHK1 regulates proliferation and survival responses in triple-negative breast cancer
Source: Oncotarget. 2014 Mar 27;5(15):5920–33. doi: 10.18632/oncotarget.1874 (PMC4171602; doi:10.18632/oncotarget.1874)
Supplement: Supplementary file 1 [file oncotarget-05-5920-s001.pdf]

SPHK1 regulates proliferation and survival responses in triple-negative breast cancer

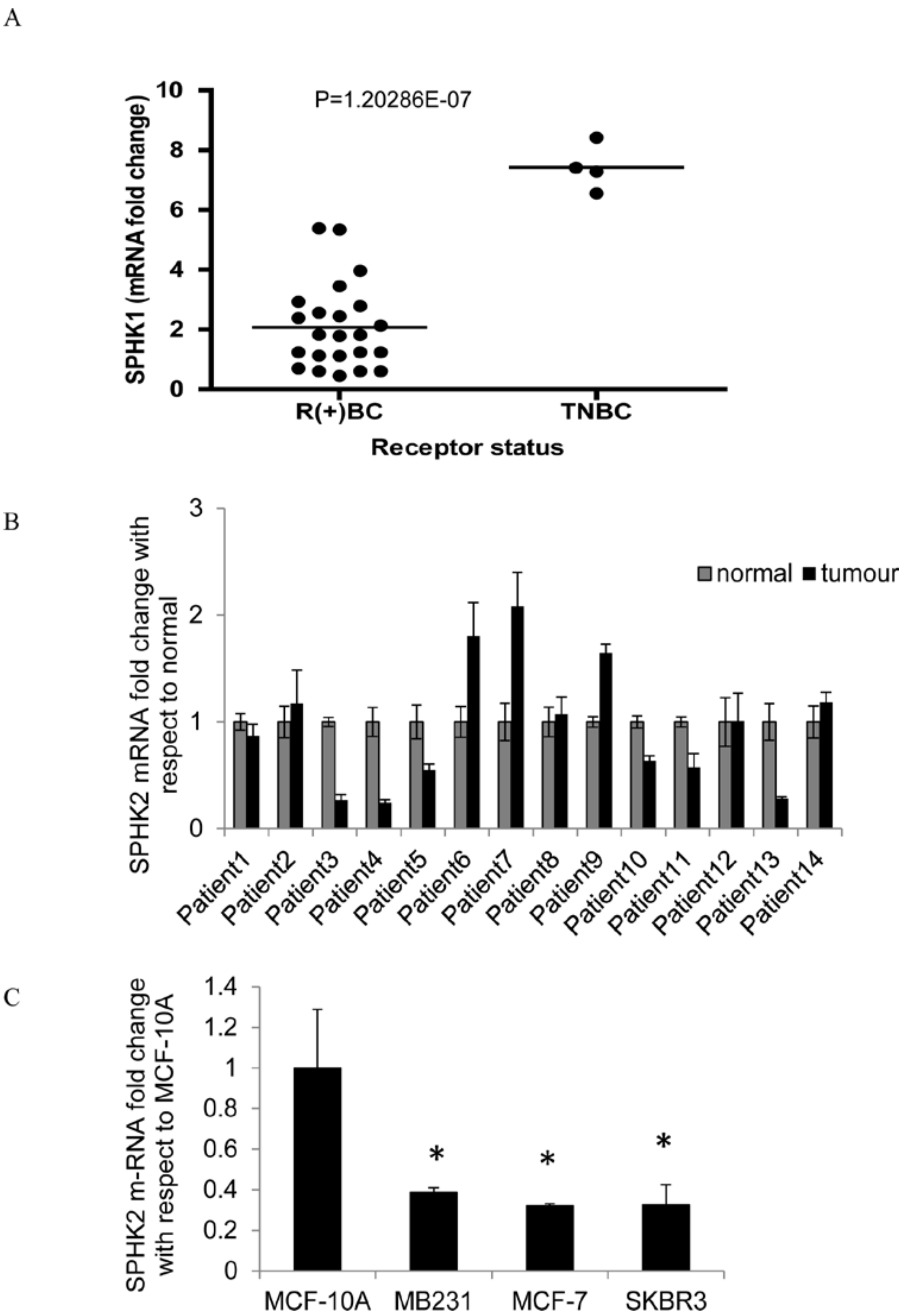

Supplementary Data Figure 1. (A) SPHK1 mRNA expression in receptor-positive, R (+), and triple-negative breast cancer (TNBC), cases. Each dot corresponds to an individual patient's fold change in relative SPHK1 mRNA levels between tumor and adjacent normal tissue. (B) The mRNA expression of SPHK2 in 14 human breast tumors and their paired adjacent normal breast

tissues was examined by real-time PCR. Expression levels were normalized with GAPDH. The level of SPHK2 mRNA for each tumor was calculated relative to that in the adjacent normal breast tissue. In the majority (78.5%) of tumors, SPHK2 mRNA levels were either reduced (6 of 14 cases) or unchanged (5 of 14 cases) compared to adjacent normal tissues. Only 3 cases exhibited increased SPHK2 mRNA expression. (C) SPHK2 mRNA level in breast cancer cell lines compared to MCF-10A. SPHK2 mRNA levels was found to be very low in breast cancer cell lines compare to MCF-10A. Statistical analysis was performed using Student's t-test. Column shows mean of 3 experiments +/- S.D. *p < 0.05.

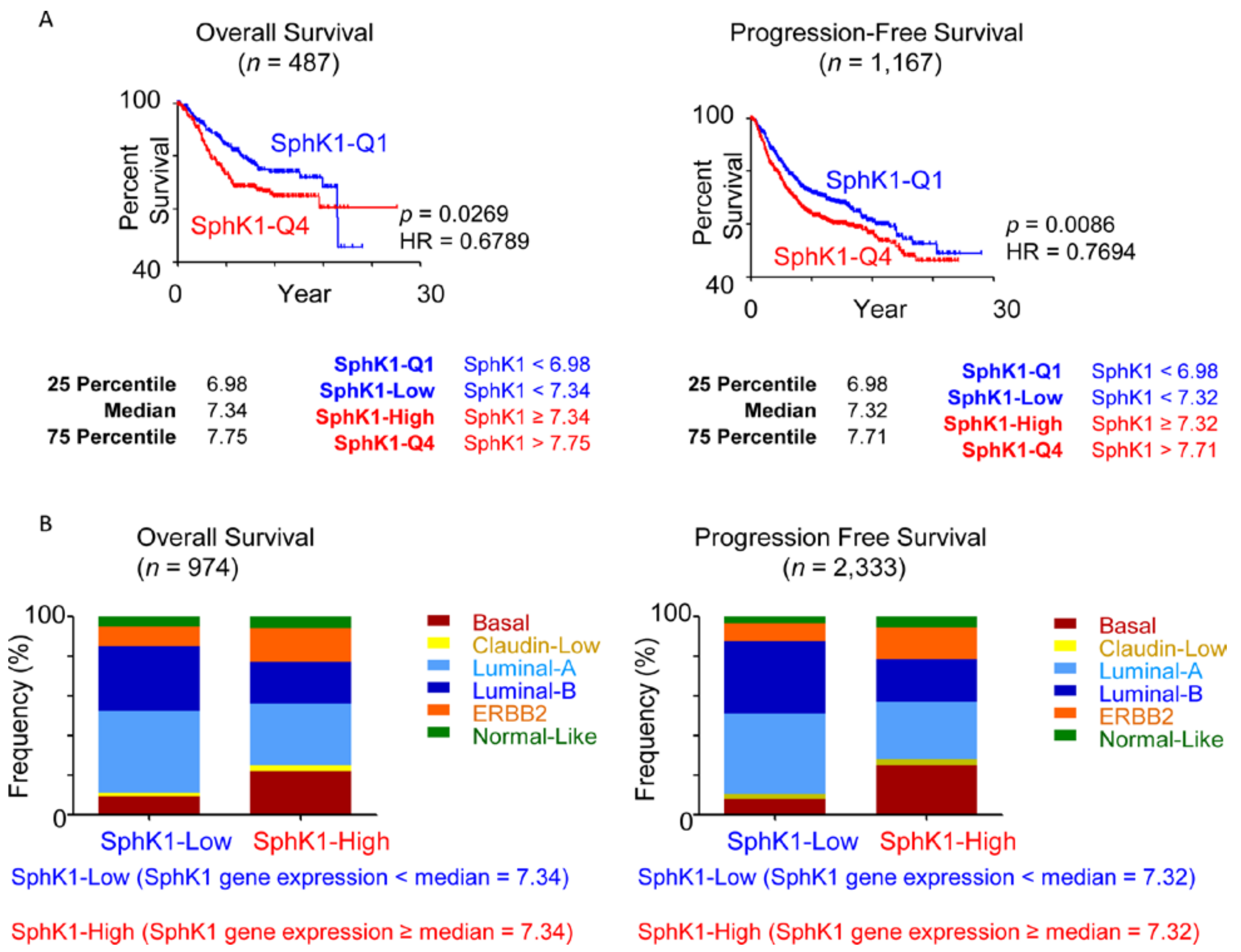

Supplementary Data Figure 2. (A) SPHK1-low and high are defined based on the median expression of SPHK1 (7.34 for OS, and 7.32 for PFS) in the breast cancer cohort we have (n = 3,992). SPHK1-low is the group of breast cancer tumors having lower expression than the median, whereas SPHK1-high is the breast cancer tumors that have higher expression than the median. (B) Subtype distribution of SphK1-low and –high groups in samples with overall survival information (left panel) and in samples with progression-free survival information (right panel). Subtype color code: Basal, maroon; Claudin-low, yellow; Luminal-A, light blue; Luminal-B, dark blue; ERBB2+, orange; Normal-like, green.

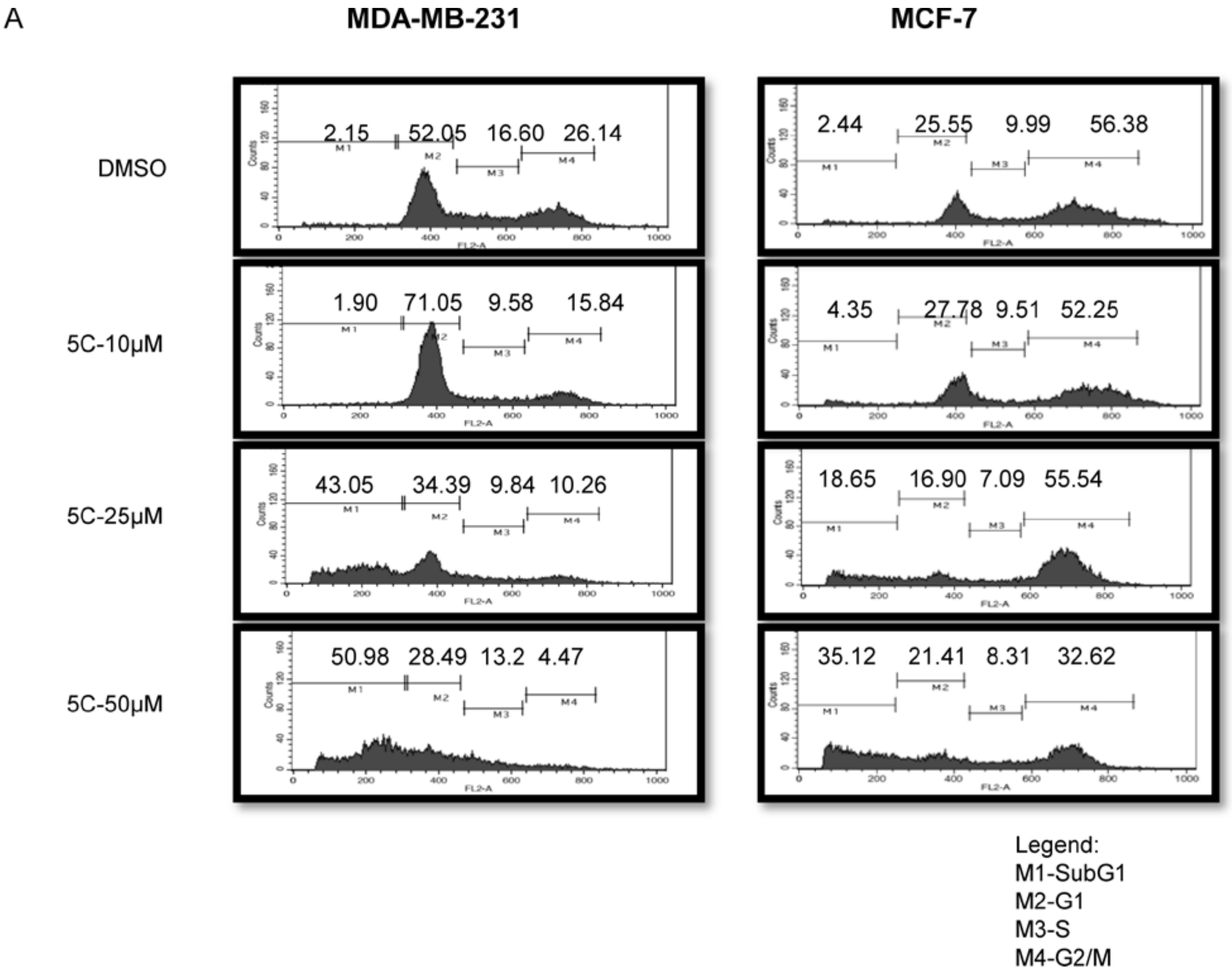

Supplementary Data Figure 3. (A) Flow cytometric analysis of PI staining in fixed MDA-MB-231 and MCF-7 cell lines after treatment with 10-50µM SKI-5C for 24 hours. This figure shows the percentage of cells in different cell cycle phases. 25-50 µM SKI-5C induced significant increases in the sub-G1 populations indicating cell death. Figures are representatives from three independent experiments.

A

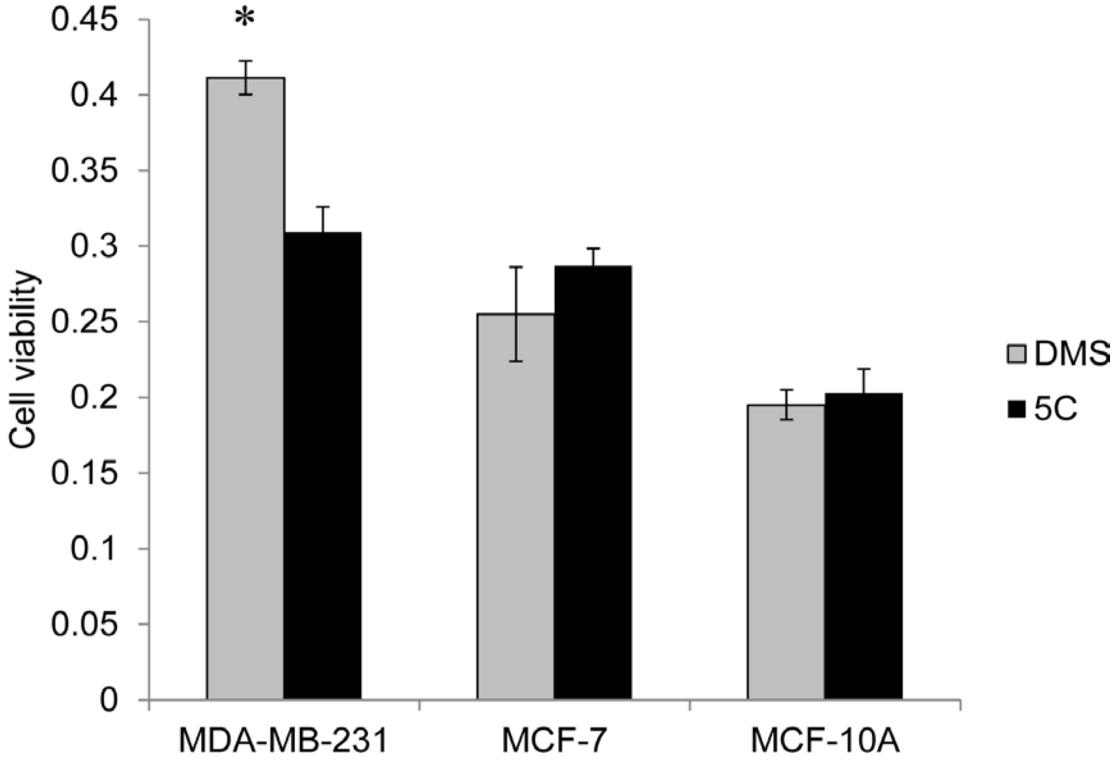

Supplementary Data Figure 4. (A) MDA-MB-231, MCF-7 and MCF-10A cells were cultured under standard conditions in the presence of 10µM SKI-5C and DMS for 24 hours. Inhibitory effects on cell growth were assessed by WST assay. Data represents the mean ± SD of at least three independent experiments; *$p < 0.05$.

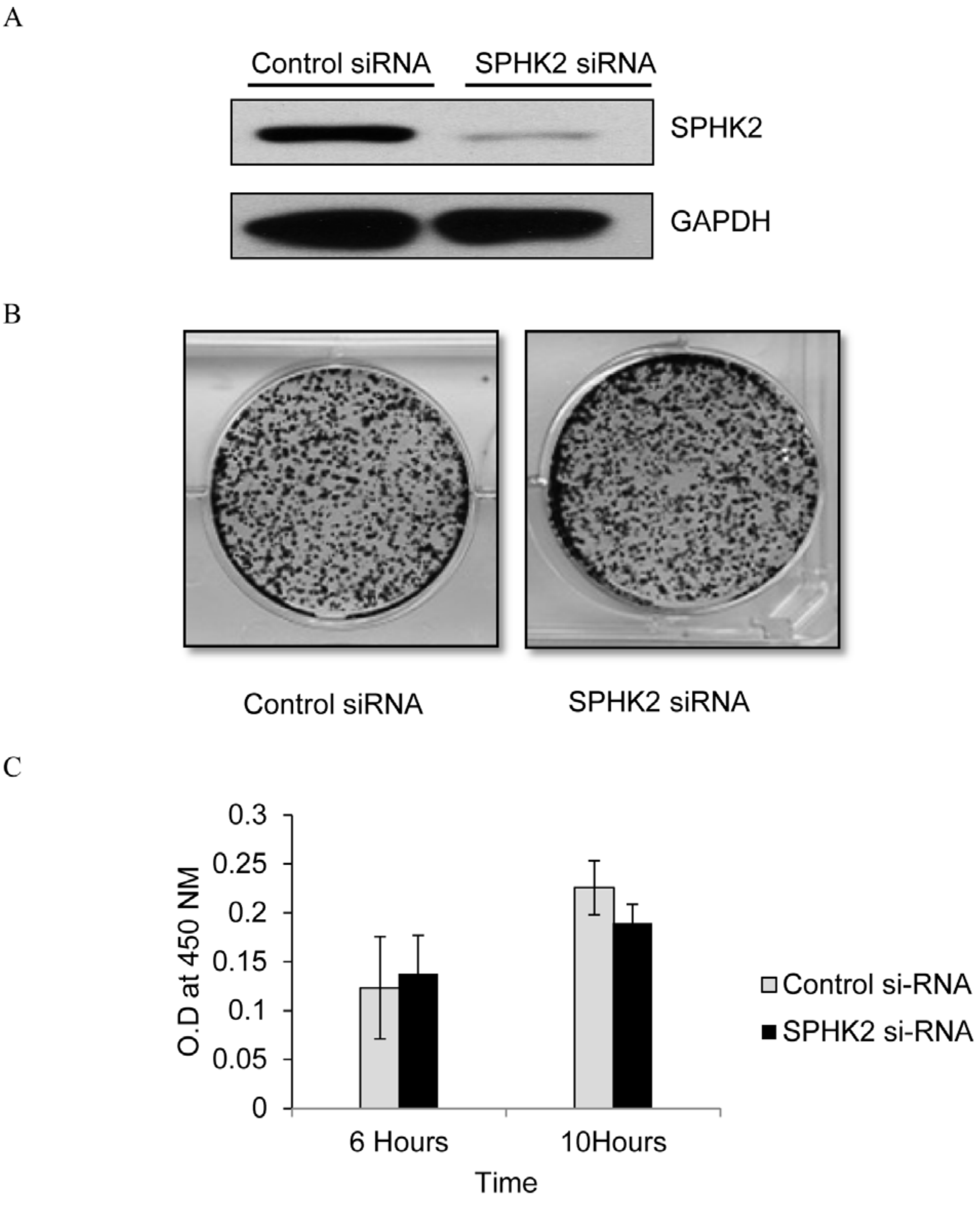

Supplementary Data Figure 5. (A) Representative western blot of SPHK2 and GAPDH in MDA-MB-231 after transfection with SPHK2-specific siRNA or control siRNA for 48 hours. (B) Colony formation assay after knock down of SPHK2 on MDA-MB-231 cells. (C) BrdU assay after knock down of SPHK2 on MDA-MB-231 cells. Data represents the mean ± SD of at least three independent experiments.

A

Drugs	Cell lines	IC-50	S.D
SKI-5C	MDA-MB-231	38.93 μM	± 4.73
Doxorubicin	MDA-MB-231	35.86 μM	± 0.058
5-FU	MDA-MB-231	72 μM	± 0.100
Docetaxel	MDA-MB-231	100 nM	± 0.0503

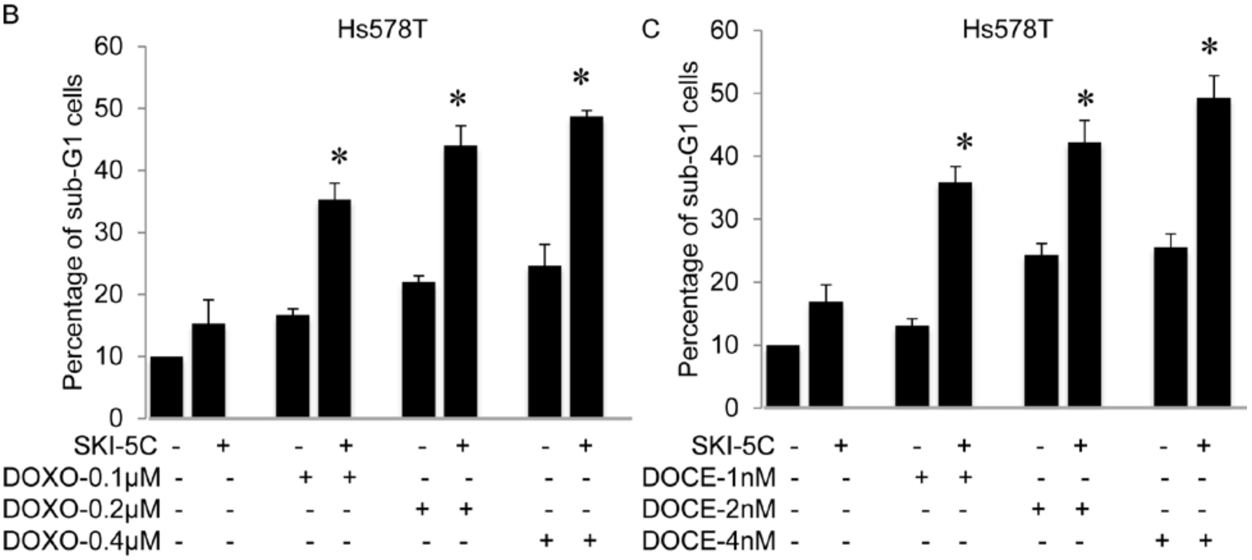

Supplementary Data Figure 6. (A) IC50 of drugs in MDA-MB-231 cells treated with SKI-5C, Doxorubicin, 5-FU and Docetaxel for 24 hours. Inhibitory effects on cell growth were assessed by the WST assay. Data represents the mean ± SD of at least three independent experiments. (B-C) Sub-G1 populations by PI analysis in combination treatment of cells using 10μM SKI-5C with low doses (below IC50) of 0.1-0.4μM Doxorubicin or 1-4nM Docetaxel for 24 hours compared to treatment with either SKI-5C, Doxorubicin or Docetaxel alone. Data represents the mean ± SD of at least three independent experiments; *$p < 0.05$.
