# Supplementary material for: SPHK1 regulates proliferation and survival responses in triple-negative breast cancer
Source: Oncotarget. 2014 Mar 27;5(15):5920–33. doi: 10.18632/oncotarget.1874 (PMC4171602; doi:10.18632/oncotarget.1874)
Supplement: Supplementary file 2 [file oncotarget-05-5920-s002.pdf]

Supplementary Data Tables

Table 1: Clinical parameters of breast cancer patients

Sample No	Anatomic Site	Age	Race	Histology	Grade	ER	PR
1.	**Breast, NOS**	**53**	**Chinese**	**Infiltrating duct carcinoma**	**2**	**+**	**-**
2.	**Breast, NOS**	**50**	**Chinese**	**Infiltrating duct carcinoma**	**2**	**-**	**+**
3.	**Breast, NOS**	**66**	**Chinese**	**Mucinous carcinoma**	**1**	**+**	**-**
4.	**Breast, NOS**	**47**	**Chinese**	**Infiltrating duct carcinoma**	**3**	**+**	**+**
5.	**Breast, NOS**	**40**	**Chinese**	**Infiltrating duct carcinoma**	**3**	**-**	**-**
6.	**Breast, NOS**	**52**	**Malay**	**Infiltrating duct carcinoma**	**2**	**-**	**+**
7.	**Breast, NOS**	**54**	**Chinese**	**Infiltrating duct carcinoma**	**3**	**+**	**+**
8.	**Breast, NOS**	**43**	**Indian**	**Infiltrating duct carcinoma**	**3**	**-**	**-**
9.	**Breast, NOS**	**55**	**Chinese**	**Infiltrating duct carcinoma**	**3**	**-**	**+**
10.	**Breast, NOS**	**55**	**Chinese**	**DCIS (high grade) with microinvasion**	**3**	**NA**	**NA**
11.	**Breast, NOS**	**67**	**Chinese**	**Infiltrating duct carcinoma**	**3**	**+**	**+**
12.	**Breast, NOS**	**52**	**Chinese**	**Infiltrating duct carcinoma**	**3**	**+**	**+**
13.	**Breast, NOS**	**43**	**Chinese**	**Infiltrating duct carcinoma**	**3**	**-**	**-**
14.	**Breast, NOS**	**66**	**Chinese**	**Infiltrating duct carcinoma**	**2**	**+**	**-**
15.	**Breast, NOS**	**57**	**Indian**	**Infiltrating duct carcinoma**	**2**	**+**	**+**
16.	**Breast, NOS**	**42**	**Malay**	**Infiltrating duct carcinoma**	**3**	**-**	**+**
17.	**Right breast**	**50**	**Chinese**	**Infiltrating duct carcinoma**	**2**	**-**	**-**
18.	**Left breast**	**50**	**Malay**	**Infiltrating duct carcinoma**	**2**	**+**	**+**
19.	**Left breast**	**50**	**Others**	**Infiltrating duct carcinoma**	**2**	**+**	**+**
20.	**Right breast**	**40**	**Indian**	**Infiltrating duct carcinoma**	**3**	**-**	**+**
21.	**Left breast**	**65**	**Chinese**	**Infiltrating duct carcinoma**	**3**	**+**	**+**
22.	**Right breast**	**57**	**Chinese**	**Infiltrating duct carcinoma**	**3**	**+**	**+**
23.	**Left breast**	**42**	**Malay**	**Infiltrating duct carcinoma**	**2**	**+**	**+**
24.	**Breast, NOS**	**41**	**Chinese**	**Infiltrating duct carcinoma**	**3**	**+**	**+**
25.	**Breast, NOS**	**47**	**Chinese**	**Infiltrating duct carcinoma**	**3**	**-**	**-**
26.	**Left breast**	**61**	**Malay**	**Infiltrating duct carcinoma**	**3**	**+**	**+**
27.	**Left breast**	**76**	**Chinese**	**Infiltrating duct carcinoma**	**3**	**+**	**+**
28.	**Left breast**	**57**	**Others**	**Infiltrating duct carcinoma**	**3**	**+**	**-**
29.	**Breast, NOS**	**64**	**Chinese**	**Infiltrating duct carcinoma**	**3**	**-**	**+**
30.	**Breast, NOS**	**58**	**Sikh**	**Infiltrating duct carcinoma**	**3**	**+**	**+**
31.	**Right breast**	**45**	**Chinese**	**Infiltrating duct carcinoma**	**3**	**+**	**+**
32.	**Right breast**	**45**	**Chinese**	**Infiltrating duct carcinoma**	**3**	**+**	**+**

Supplementary Data Tables

Table 2: Normalized mean and median values of SPHK1 expression in breast cancer subtypes from microarray data

Parameter\Subtype	Basal	ClaudinLow	LuminalA	LuminalB	ERBB2	NormLike
Mean	7.872	7.56	7.247	7.195	7.505	7.477
Lower 95% CI of mean	7.814	7.442	7.221	7.166	7.459	7.416
Upper 95% CI of mean	7.929	7.677	7.274	7.224	7.551	7.538
SEM	0.02923	0.05928	0.01359	0.01467	0.02346	0.03102
Median	7.817	7.503	7.238	7.186	7.47	7.452

Table 3: Pair-wise Comparison of SPHK1 expression (Mann Whitney-U test/MW) MW. p values

MW.p-value	Basal	ClaudinLow	LuminalA	LuminalB	ERBB2	NormLike
Basal	1	4.17E-06	5.40E-77	7.83E-86	5.21E-18	5.60E-13
ClaudinLow		1	5.73E-07	3.33E-09	0.866975	0.528201
LuminalA			1	0.008838	1.23E-21	1.03E-09
LuminalB				1	1.71E-29	5.09E-14
ERBB2					1	0.429359
NormLike						1
